# Metabolic profiling in experimental guinea pig models of bacterial and allergic inflammation

**DOI:** 10.1007/s11306-025-02239-x

**Published:** 2025-03-23

**Authors:** J. Hanusrichterova, E. Baranovicova, R. Barosova, M. Kolomaznik, P. Mikolka, P. Kosutova, D. Mokra, J. Mokry, A. Calkovska

**Affiliations:** 1 Biomedical Centre Martin, Jessenius Faculty of Medicine in Martin, Comenius University in Bratislava, Mala Hora 11161/4D, 03601 Martin, Slovakia; 2 Department of Physiology, Jessenius Faculty of Medicine in Martin, Comenius University in Bratislava, Mala Hora 11161/4C, 03601 Martin, Slovakia; 3 Department of Pharmacology, Jessenius Faculty of Medicine in Martin, Comenius University in Bratislava, Mala Hora 11161/4C, 03601 Martin, Slovakia

**Keywords:** Bacterial lipopolysaccharide, Ovalbumin, Allergy, Inflammation, Metabolomics

## Abstract

**Introduction:**

Based on distinct triggers, bacterial and allergen-induced inflammatory reactions have different pathophysiology. Metabolomic analysis is high-throughput technique that can provide potential biomarkers to distinguish between these responses.

**Objectives:**

In order to find out the metabolic profiles of two types of inflammation, metabolites were analysed in blood plasma and bronchoalveolar lavage fluid (BALF) of guinea pigs subjected to bacterial lipopolysaccharide (LPS) or allergen ovalbumin (OVA).

**Methods:**

Hydrogen-1 nuclear magnetic resonance (^1^H NMR) spectroscopy for metabolite analysis was performed in samples of blood plasma and BALF of guinea pigs.

**Results:**

Random forest algorithm built on combination of levels of circulating and BALF metabolites resulted in almost ideal discrimination between acute allergic and bacterial inflammation. The differences between inflammation triggered by LPS and OVA were manifested in shift in energy metabolism, metabolism of branched-chain amino acids (BCAAs)/branched-chain keto acids (BCKAs) with alterations in alanine and glutamine, which are linked with both, ammonia homeostasis as well as gluconeogenesis.

**Conclusion:**

Distinct molecule nutrients are to be utilized during acute bacterial and allergic inflammatory response.

**Supplementary Information:**

The online version contains supplementary material available at 10.1007/s11306-025-02239-x.

## Introduction

Respiratory system is often exposed to Gram-negative bacterial cell wall component lipopolysaccharide. In plasma, LPS binds to LPS-binding protein (LPB) and activates Toll-like receptor 4 (TLR4). TLR4 associates with MD-2 (lymphocyte antigen 96) and membrane protein CD14 to form an active signalling complex (Pallett et al., 2023). LBP and CD14 are important proteins in LPS activation of different cell populations, including neutrophils, macrophages, and endothelial cells. LPS is profound mediator of neutrophil activation and infiltration into infected tissues (Soler-Rodriguez et al., 2000). TLR4 activates two distinct inflammatory pathways: 1. MyD88-dependent pathway leading to activation of NF-κB and pro-inflammatory chemokine release and 2. MyD88-independent pathway leading to the production of type 1 interferons (Vadiveloo et al., 2000; Juskewitch et al., 2012; Karimi et al., 2017). Ovalbumin, chicken egg albumin, leads to allergic inflammation of the airways with an increase in eosinophil population and airway reactivity (Insuela et al., 2019). OVA-induced inflammation is characterized by a type 2 immune response involving eosinophils, type 2 helper T (Th2) cells, group 2 innate lymphoid cell (ILC2) influx, release of type 2 cytokines including IL-4, IL-5, and IL-13 (Warren et al., 2019) characteristic for allergic asthma. Ovalbumin exposure leads to ASM thickening, extracellular matrix deposition, goblet cell hyperplasia and mucus plug formation (Subhashini et al., 2016; Casaro et al., 2019). Both inflammatory triggers, LPS and OVA, have been used in our experiments to induce acute systemic and lung inflammation. Metabolomics is a powerful tool for the analysis of functional metabolites (small molecules < 1500 Da) (Jutley & Young, 2015) or biomarkers to understand physiological and pathophysiological mechanisms in a given condition of biological system (Daley-Yates et al., 2022). Metabolite levels are modified by inflammation and may predict the severity and progress of the disease as well the therapy options (Jutley & Young, 2015). The study aimed to identify significant metabolite changes during bacterial and allergen-induced acute inflammation of guinea pigs as potential markers for evaluation of ongoing inflammation and its progress.

## Materials and methods

The design of the study was approved by the local Ethics Committee of the Jessenius Faculty of Medicine, Comenius University in Martin (permission no. EK 46/2018) and the State Veterinary and Food Administration of the Slovak Republic.

## Experimental animals

Twenty-eight male adult guinea pigs (Dunkin-Hartley; 355 ± 93 g of body weight) were obtained from the Central Animal Facility of the Jessenius Faculty of Medicine in Martin, Slovakia and the breeding station Velaz, Ltd. (Prague, Czech Republic), kept in the faculty animal facility during 5 days quarantine and further 4- (LPS protocol) or 14-day (OVA protocol) sensitization period at standard conditions (12 h light/dark cycle, temperature 21 ± 2 °C, humidity 55 ± 10%, and food and water at their disposal). Guinea pigs exhibit a robust immune response that closely mimics human hypersensitivity reactions. Their larger size allows for easier handling and more significant sampling, which can facilitate more detailed analyses of immune responses compared to smaller animals like mouse (Maurer, 2007; Kamata et al., 2022).

## OVA sensitization

**Fig. 1:**
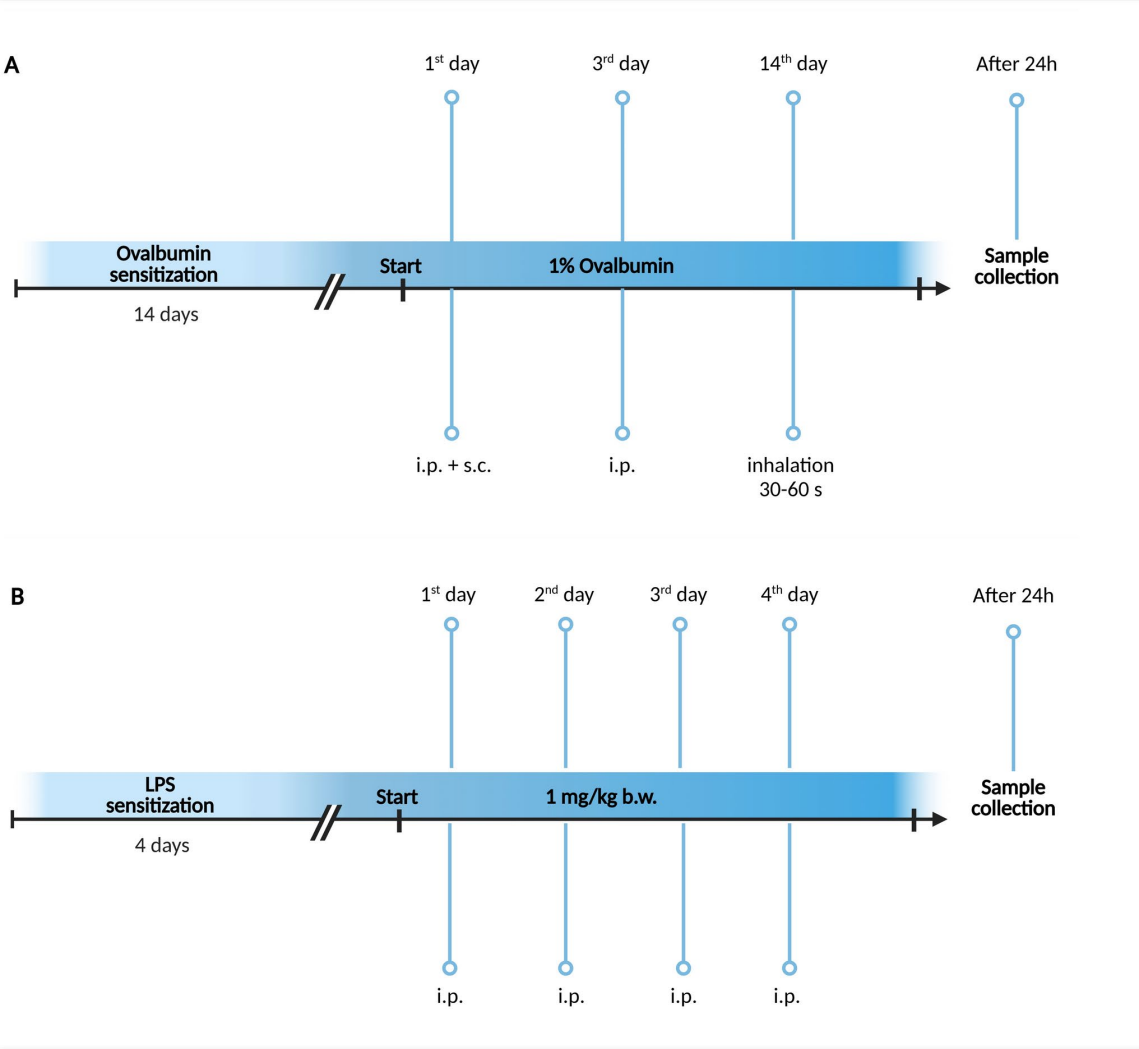
OVA (**A**) and LPS (**B**) sensitization schemes. Created with BioRender.com

The animals were sensitized with OVA (Sigma-Aldrich, Inc., Saint-Louis, Missouri, United States) according to previously used protocol (Mokry et al., 2008). The animals in the group OVA (n = 5) were injected intraperitoneally (0.5 mL) and subcutaneously (0.5 mL) with 1% OVA solution supplemented with 0.1% of adjuvant (aluminum hydroxide) on day 1 and intraperitoneally (1 mL) on day 3. On day 14, the animals inhaled aerosol of 1% OVA in sterile saline for 30–60 s (B. Braun company, Melsungen AG, Germany). The animals were sacrificed by lethal dose of anaesthetics and experiments were performed 24 h after the last inhalation with OVA (Fig. 1A). Control group (n = 8) did not receive any treatment.

## LPS sensitization

The animals (n = 7) were sensitized by intraperitoneal (i.p.) injection of LPS from *Escherichia coli* O55:B5 (Sigma-Aldrich, Inc., Saint-Louis, Missouri, United States) at a dose 1 mg/kg b.w. for 4 days, once a day (group LPS) (Yamawaki et al., 1990) (Fig. 1B). Control group (n = 8) did not receive any treatment.

## Blood plasma and BALF sample collection

The animals were sacrificed by intraperitoneal injection of tiletamine and zolazepam at the dose 150 mg/kg of b.w. (Zoletil, Virbac SA, Carros, France) combined with xylazinum anaesthetics at the dose of 50 mg/kg of b.w. (Xylariem, Ecuphar N.V., Oostkamp, Belgium) 24 h after the last OVA inhalation or LPS intraperitoneal injection (one day post-quarantine in control animals). Blood was collected after the heart puncture and BAL fluid was recovered from left lung lobes after double lavage with the saline solution heated to 37 °C at the dose of 10 mL/kg b.w.. Blood samples were immediately centrifuged at 590 × *g* for 15 min at 4 °C and plasma supernatants were stored at − 80 °C until metabolomic assay. After the lavage, BALF samples were immediately centrifuged at 600 × *g* for 15 min at 4 °C and supernatants were stored at − 80 °C until metabolomic assay.

## Hydrogen-1 nuclear magnetic resonance (^1^H NMR) spectroscopy analysis

### ***Samples preparation for ***^***1***^***H NMR analysis***

Plasma was deproteinized according to Nagana Gowda et al. (2015) as follows: the mixture obtained after adding 600 μl of methanol to 300 μl of plasma was shortly vortexed and stored at − 20 °C for 20 min. After centrifugation at 14,000 rpm (14,800 × *g*), 15 min, 650 μl of supernatant were dried out. Before measurement, the dried matter was carefully mixed with 500 μl of deuterated water and 100 μl of stock solution (100 mM phosphate buffer, pH-meter reading 7.40, and 0.25 mM of 3-(trimethylsilyl)-propionic-2,2,3,3-d_4_ acid sodium salt (TSP-d_4_) as a chemical shift reference in deuterated water). For measurement, 550 μl of the final mixture was transferred into a 5 mm NMR tube. BALF samples: 500 μl was dried out, mixed with 500 μl of deuterated water and 100 μl of stock solution. Resulting solution was transported into a 5 mm NMR tube.

### ^***1***^***H NMR data acquisition***

NMR data were acquired using 600 MHz NMR spectrometer (Avance III, Bruker, Germany) equipped with triple resonance (TCI) cryoprobe. Initial settings were performed on an independent sample and adopted for measurements. Samples were stored in Sample Jet at approximately 6 °C before measurement. Bruker profiling protocols were modified as follows: profiling 1D NOESY with presaturation (noesygppr1d): FID size 64k, dummy scans 4, number of scans 128, spectral width 20.4750 ppm; COSY with presaturation was acquired for randomly chosen 10 samples (cosygpprqf): FID size 4k, dummy scans 8, number of scans 1, spectral width 16.0125 ppm; homonuclear J-resolved (jresgpprqf): FID size 8k, dummy scans 16, number of scans 4; profiling CPMG with presaturation (cpmgpr1d, L4 = 126, d20 = 3 ms): FID size 64k, dummy scans 4, number of scans 256 for blood plasma and 1024 for BALF, spectral width 20.0156 ppm. All experiments were conducted with a relaxation delay of 4 s; all data were once zero-filled. An exponential noise filter was used to introduce 0.3 Hz line broadening before the Fourier transformation. Samples were measured at 310 K and randomly ordered for acquisition.

### ^***1***^***H NMR data analysis***

A chemical shift of 0.000 ppm was assigned to TSP-d_4_ signal. All spectra were binned to bins of the size of 0.001 ppm, starting from 0.500 ppm to 9.500 ppm. No normalization method was applied on NMR data of blood plasma, as exactly the same amount of blood plasma was taken from all samples. NMR data of BALF were normalized to the sum before quantitative evaluation. Spectra were solved using internal metabolite database, online human metabolome database (www.hmdb.ca) (Wishart et al., 2022), chenomx software free trial version and literature (Nagana Gowda et al., 2015). For all compounds the multiplicity of peaks was confirmed in J-resolved spectra and homonuclear cross peaks were confirmed in cosy spectra. For the list of chemical shifts, J couplings and multiplicities see Online Supplement (Suppl. Tab. S1). After the metabolites were identified we chose the spectra subregions with only single metabolite assigned. In 0.001 ppm binned spectra we summed integrals of selected signals. These values were handled as relative concentrations of metabolites in the samples. Metabolites not having appropriate signals for the evaluations or with unambiguous peak assignment were excluded from further evaluation. Note: in this work we use the trivial names of 2-oxoisocaproate—ketoleucine, 3-methyl-2-oxovalerate—ketoisoleucine and 2-oxoisovalerate—ketovaline to better evoke the origin of the ketoacids.

### Statistical analysis

The null hypothesis of equality of population medians among groups was tested by the non-parametric Mann–Whitney U test (Matlab v2018b), and medians were used to calculate the percentual changes. PCA (Principal component analysis), PLS-DA (Partial least squares-discriminant analysis) (Lê Cao et al., 2011) and cross validated data discrimination based on random forest algorithm were performed using online tool Metaboanalyst v6.0, accessed in 01-02/2024 (Xia et al., 2015).

## Results

Differential white blood cell count was classified from samples of whole blood and BALF immediately after collection and increased neutrophils were found in BALF after LPS sensitization (vs. Control, *p* < 0.01) and increased eosinophils were found in BALF after OVA sensitization (vs. Control, *p* < 0.01) (Suppl. Fig. S1).

## Metabolomic profile of blood plasma after LPS and OVA sensitization

### Multivariate and discriminative data analysis of blood plasma

**Fig. 2:**
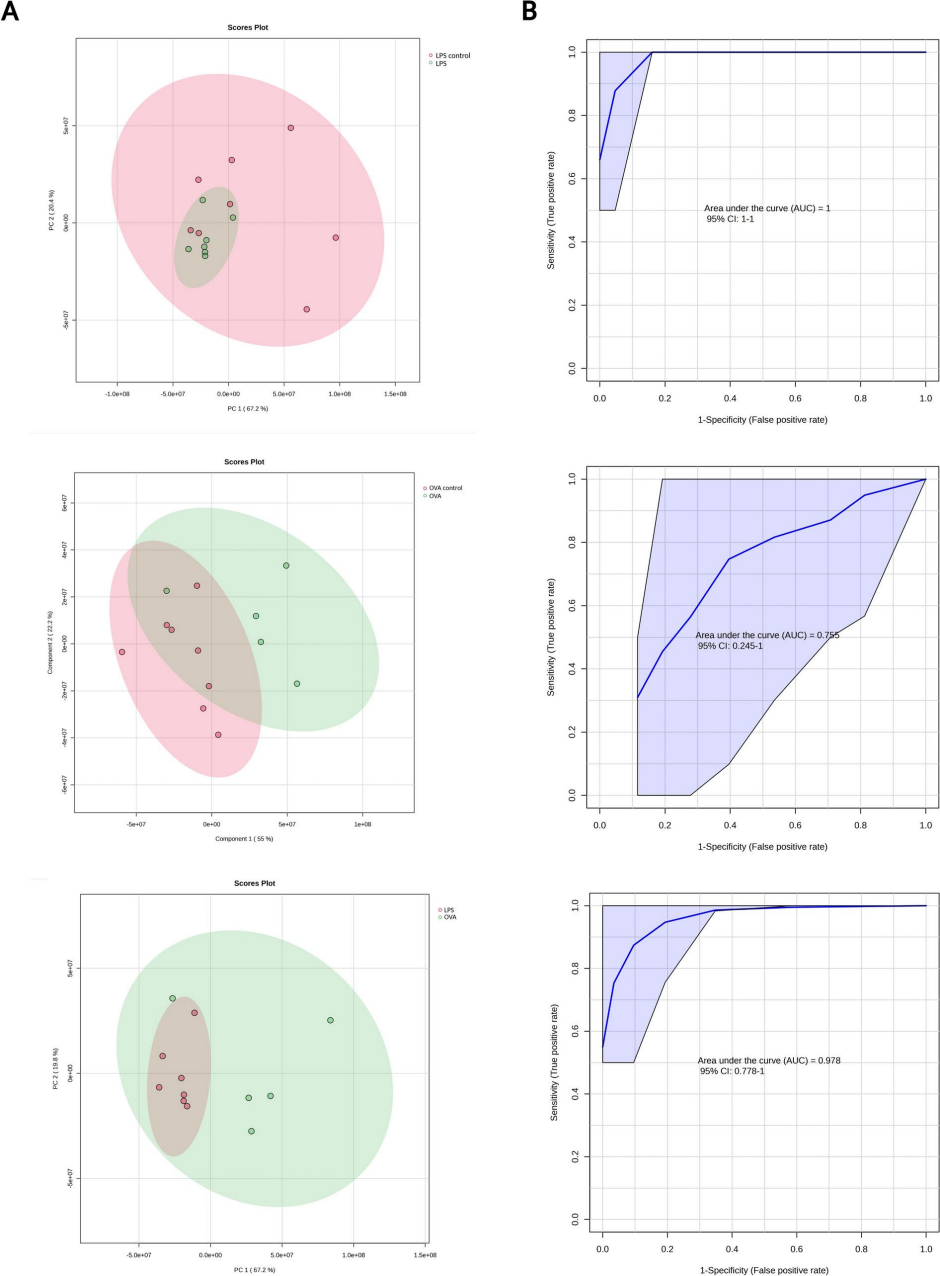
PCA **A** analysis of plasmatic metabolome for groups LPS control and LPS, OVA control and OVA and LPS and OVA with 95% confidence ellipse (ellipse that defines the region that contains 95% of all samples). As input variables, relative levels of blood plasma metabolites were used. **B**: ROC curves derived from random forest discrimination, displayed for 3 features of the highest importance, relative levels of blood plasma metabolites were used as input variables (Color figure online)

Supervised PCA analysis visualizes metabolic data in clusters, indicating the variability or similarity within and between groups. LPS sensitization led to a reduction in data fluctuation when compared not only to control group (Fig. 2A above), but also when comparing to OVA sensitization group (Fig. 2A below), what may indicate consistent metabolic changes. The variability of metabolic data from OVA sensitization was comparable to those of adequate controls (Fig. 2A middle), with partially overlapping clusters. Multivariate PLS-DA confirmed the above descripted trends (Suppl. Fig. S2A). The included discriminatory algorithm caused the greater shift of ellipsoids from each other, however still remaining partially overlapped (Suppl. Fig. S2A). For estimation more realistic discriminatory power of the system, we employed random forest algorithm, which is, in comparison to PLS-DA, not known to overfit the data. The algorithm achieved very good discrimination for binary systems: OVA-Control: AUC = 0.755 and predictive accuracy 70.30% for glucose, 3-hydroxy-butyrate and valine, further for LPS-Control: AUC = 1 and predictive accuracy 99.60% for pyruvate, histidine and glutamine, and for LPS-OVA: AUC = 0.978 and predictive accuracy 99% for alanine, pyruvate and histidine. More detailed results from PCA, PLS-DA and RF are available in the Online Supplement file (Suppl. Tab. S4).

**Fig. 3:**
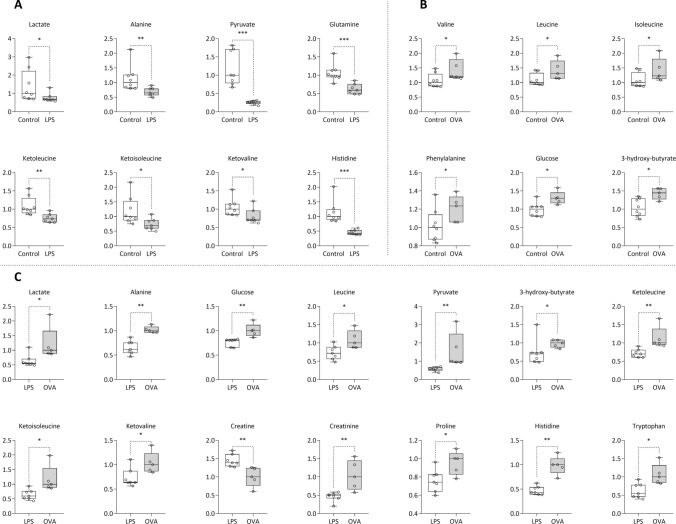
Relative concentrations of plasma metabolites after 4-day LPS sensitization (**A**) and 14-day sensitization with OVA (**B**) and comparison between LPS and OVA (**C**), data normalized to median of control groups (**A**, **B**) or OVA (**C**) set to 1. LPS control n = 8; LPS n = 7; OVA control n = 8; OVA n = 5. Line at the bar represents the median. Data compared using non-parametric Mann–Whitney U test. Statistical significance *p < 0.05; **p < 0.01; ***p < 0.001

In the plasma of animals sensitized with lipopolysaccharide, we observed significantly lower levels of several metabolites, including lactate, alanine, pyruvate, glutamine, ketoleucine, ketoisoleucine, ketovaline, and histidine (*p* < 0.05–0.001) (Fig. 3A and Suppl. Tab. S2). In contrast, guinea pigs sensitized with ovalbumin exhibited significantly higher levels of metabolites such as valine, leucine, isoleucine, phenylalanine, glucose, and 3-hydroxy-butyrate in plasma (*p* < 0.05) (Fig. 3B and Suppl. Tab. S2). The similar directional pattern of metabolite changes observed in blood plasma after LPS (decrease compared to control) (Fig. 3A) and OVA (increase compared to control) (Fig. 3B), we consider as a random effect, as this trend was not evident for all other evaluated metabolites. We compared the plasma metabolite levels following LPS administration to those observed after OVA sensitization. The analysis showed significantly lower levels of several metabolites, including lactate, alanine, glucose, leucine, pyruvate, 3-hydroxy-butyrate, ketoleucine, ketoisoleucine, ketovaline, creatinine, proline, histidine, and tryptophan (*p* < 0.05–0.01). In contrast, creatine levels were significantly higher (*p* < 0.01) in the LPS group compared to the OVA group (Fig. 3C).

## Metabolomic profile of BALF after LPS and OVA sensitization

### Multivariate and discriminative data analysis of BALF

**Fig. 4:**
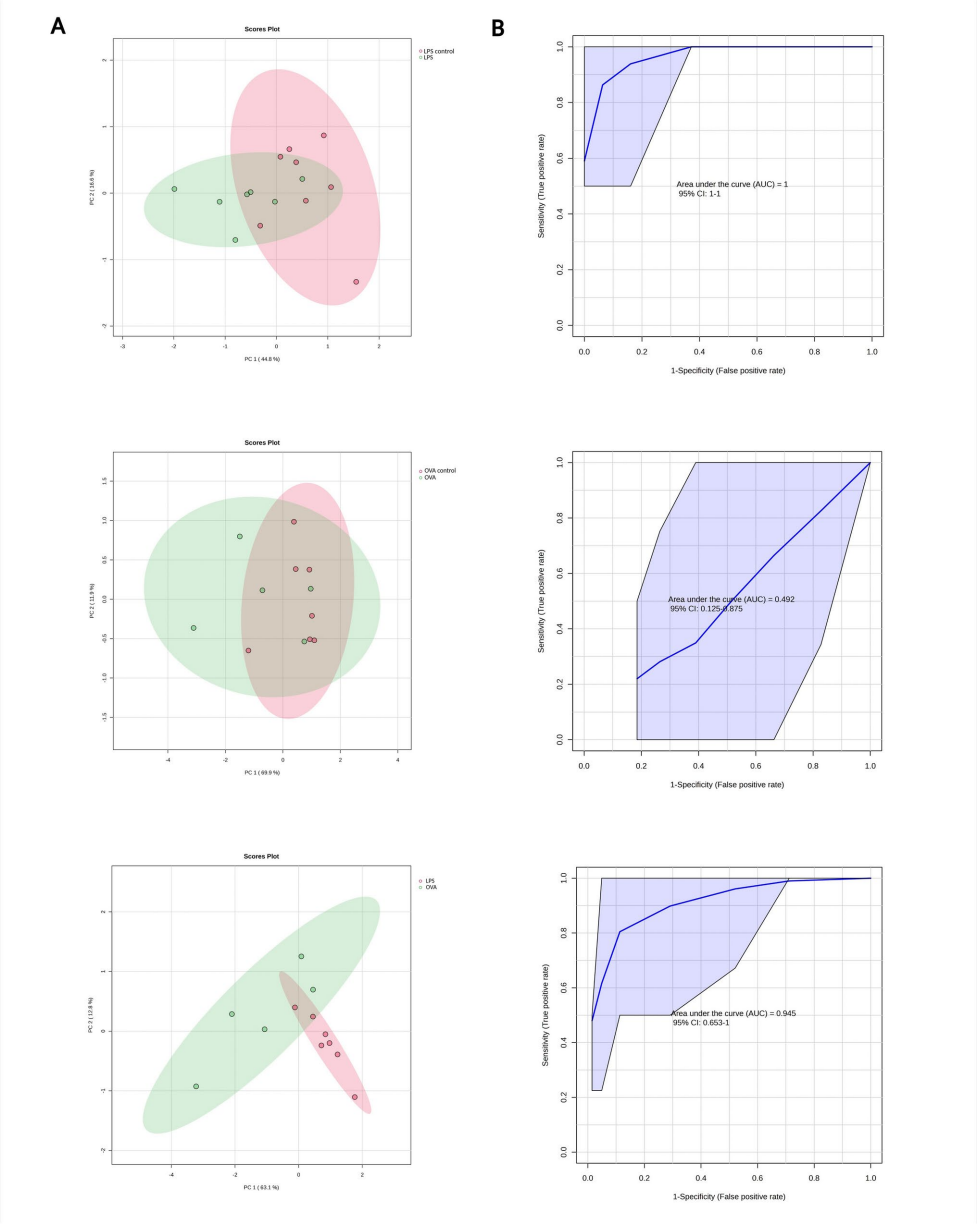
PCA **A** analysis of BALF metabolome for groups LPS control and LPS, OVA control and OVA and LPS and OVA with 95% confidence ellipse (ellipse that defines the region that contains 95% of all samples). As input variables, relative levels of BALF metabolites were used. **B**: ROC curves as a result from random forest discrimination displayed for 3 features of the highest importance, relative levels of BALF metabolites were used as input variables (Color figure online)

In comparison to blood plasma metabolome, the LPS sensitization induced metabolic changes in BALF were not manifested in reduced data variability in LPS animals in comparison to controls (Fig. 4A above), however, recognizable reduced data variation in LPS guinea pigs was observed in comparison with OVA group (Fig. 4A below). As expected, PLS-DA analysis enhanced the clusters separations, where for LPS-Control and LPS-OVA systems, 95% confidence ellipsoids were well separated from each other (Suppl. Fig. S2B). RF discrimination yielded following results: LPS-Control: AUC = 1 and predictive accuracy 90.80% for pyruvate, succinate and alanine, OVA-Control: AUC = 0.492 which is of no practical use, and LPS-OVA: AUC = 0.945 and predictive accuracy 81.70% for succinate, tyrosine and alanine. More detailed results from PCA, PLS-DA and RF are available in the Online Supplement file (Suppl. Tab. S4).

**Fig. 5:**
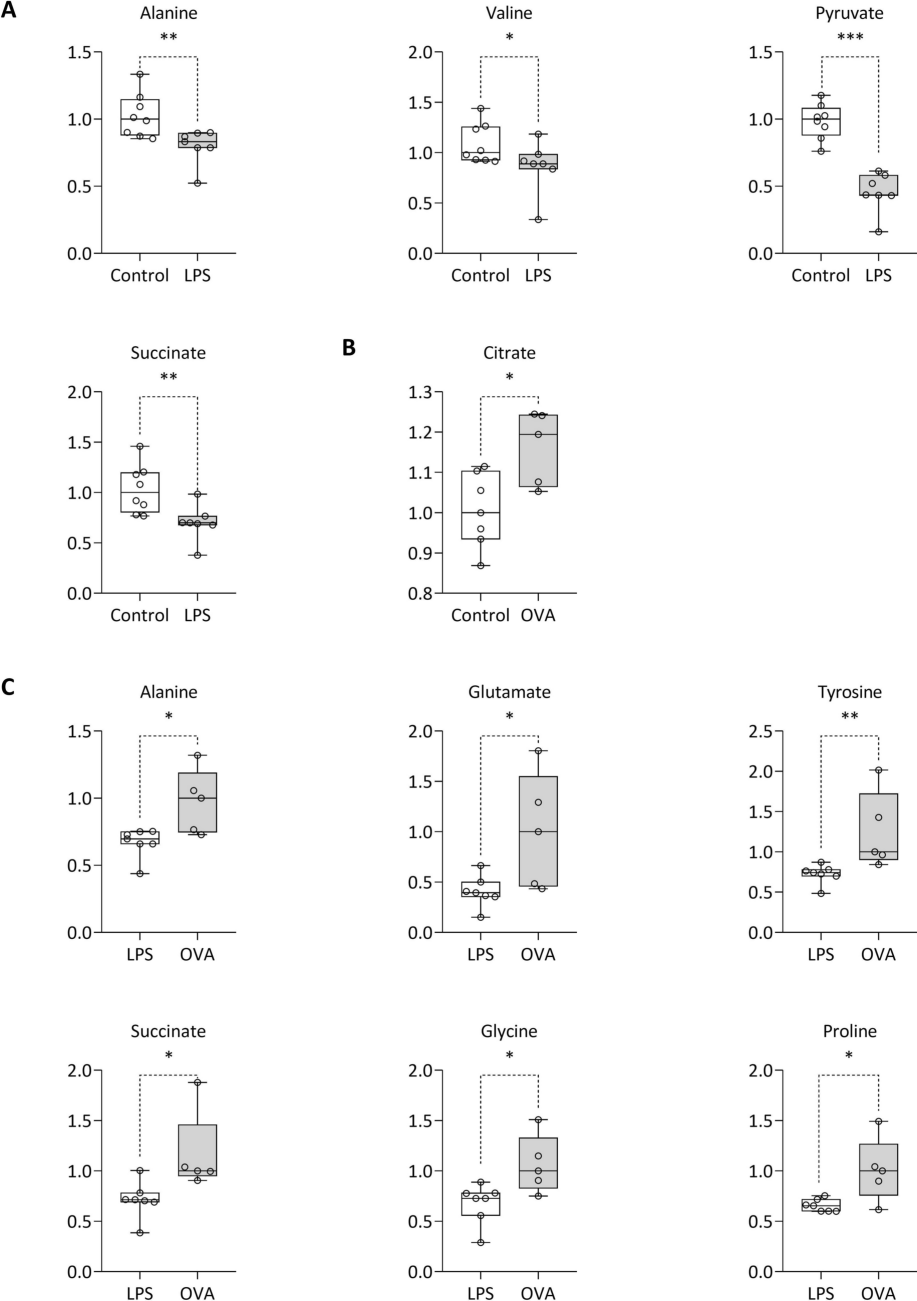
Relative concentrations of BALF metabolites after 4-day LPS sensitization (**A**) and 14-day sensitization with OVA (**B**) and comparison between LPS and OVA (**C**), data normalized to median of control groups (**A**, **B**) or OVA (**C**) set to 1. LPS control n = 8; LPS n = 7; OVA control n = 7; OVA n = 5. Line at the bar represents the median. Data compared using non-parametric Mann–Whitney *U* test. Statistical significance *p < 0.05; **p < 0.01; ***p < 0.001

In BALF of LPS sensitized guinea pigs were found decreased metabolites as follows: alanine, valine, pyruvate, and succinate (*p* < 0.05–0.001) (Fig. 5A and Suppl. Tab. S3). In BALF of OVA sensitized guinea pigs were found increased levels of citrate compared to the Control group (*p* < 0.05) (Fig. 5B and Suppl. Tab. S3). The similar directional pattern of metabolite changes observed in BALF after LPS (decrease compared to control) (Fig. 5A), we consider as a random effect, as this trend was not evident for all other evaluated metabolites. We compared BALF metabolite levels following LPS administration to those observed after OVA sensitization (Fig. 5C). The analysis showed significantly lower levels of several metabolites, including alanine, glutamate, tyrosine, succinate, glycine, and proline (*p* < 0.05–0.01).

### Multivariate and discriminative data analysis of merged BALF and plasma metabolic data for OVA and LPS

**Fig. 6:**
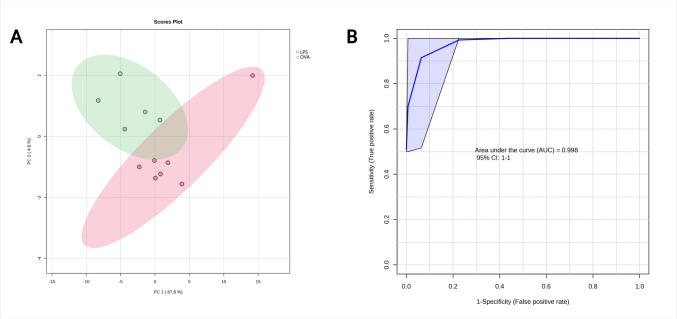
PCA **A** analysis of LPS (red) vs. OVA (green) and **B**: ROC curve as a result from random forest discrimination for 10 most important variables. For both algorithms, relative levels of metabolites in blood plasma and BALF were used as input variables (Color figure online)

When metabolomic profiles of blood plasma and BALF were combined, PCA analysis yielded two largely separated clusters (Fig. 6). The PLS-DA performed very similarly, with enhanced ellipsoids separation (Suppl. Fig. S3). Results from RF indicate almost ideal discrimination with AUC of 0.998 and oob error of 0. More detailed results from PCA, PLS-DA and RF are available in the Online Supplement file (Suppl. Tab. S4).

## Discussion

Inflammation is a physiologic response of the body to cellular or tissue damage caused by pathologic stimuli as infection, ischemia, or trauma. Metabolic activity changes rapidly during inflammation due to the recruitment of inflammatory cells and lymphocyte proliferation. Shifts in energy and oxygen demands of the inflamed tissues may result in metabolic acidosis and hypoxia. Changes in tissue metabolism include local depletion of nutrients, increased oxygen consumption following hypoxia and further lactate accumulation (Kominsky et al., 2010). During infection demands for oxygen and ATP exceed the cellular supply and lactate production increases. In the presence of oxygen, pyruvate from glucose enters mitochondria and is converted to acetyl coenzyme A, a substrate for the TCA (tricarboxylic acid or Krebs) cycle during oxidative phosphorylation. In anaerobic situation, pyruvate is directly reduced to lactate during glycolysis. The immune cells are highly metabolically active, which is especially evident during infection when they generate sufficient energy and biomolecules for growth, proliferation, and the production of pro-inflammatory molecules (Gaber et al., 2017). The primary metabolic effect of LPS is increasing lipolysis and muscle insulin resistance. Systemic cytokine and stress hormone release induce secondary insulin resistance leading to glucose intolerance and diabetes (Buhl et al., 2013). The increased TCA cycle activity noted at early time points (e.g., 8 h post-LPS stimulation) indicates heightened mitochondrial activity in response to inflammatory stimuli. However, the lack of uptake of extracellular amino acids, particularly BCAAs, suggests that despite increased energy demands, cells may not utilize BCAAs as a fuel source during early inflammation. This could imply a metabolic shift where glucose remains the primary energy substrate, potentially limiting the availability of BCAAs for conversion to keto acids (Ko et al., 2020). In our study, keto acid derivatives of BCAAs—ketoleucine, ketoisoleucine, and ketovaline were decreased in plasma and essential BCAA valine was decreased in BALF of LPS sensitized animals. Succinate levels have been found to be decreased in BALF after LPS exposure in our study. Succinate, a key metabolite in the TCA cycle and an important signalling molecule during inflammation, could fluctuate and potentially deplete following 4-days long LPS exposure as it is utilized for energy production and immune signalling. It was reported that abundance of succinate peaked at approximately 8 h post-LPS exposure of murine macrophages cells (Jiang et al., 2022). We observed decreased levels of plasmatic glutamine after LPS in our study. Endogenous glutamine synthesis does not meet the human body’s demands in catabolic conditions, such as in cancer, sepsis, infections, surgeries, traumas, as well as during intense and prolonged physical exercise. Glutamine is the most abundant amino acid in the body in health or disease and its rate of consumption by immune cells is similar or higher than that of glucose (Cruzat et al., 2018). Glutamine is released from the lungs during the early stages of endotoxin-induced injury and sepsis, which helps maintain amino acid pools in the body. However, glutamine outflow from the lungs tends to diminish in patients with adult respiratory distress syndrome, which may be caused by increased consumption by injured lung cells (Hou et al., 2012). Pyruvate has been also reduced in animals with LPS challenge. The low pyruvate levels in cells are due to reduced production and excessive consumption (Zhang et al., 2019). Pyruvate produced in the activated glycolysis is taken up by mitochondria and converted to acetylcoenzyme A by pyruvate dehydrogenase (PDH) or it is converted to lactate by lactate dehydrogenase (LDH) in the cytosol (Takeda et al., 2022). The cancer cells rely on aerobic glycolysis to generate enough energy and intermediates. This Warburg effect was described in cancer cells and converts the glycolysis-induced pyruvate into lactate making low pyruvate status in cells (Warburg, 1956). Plasmatic lactate was also decreased after LPS sensitization in our experiments. Blood lactate concentration is a balance between lactate production and uptake in organs (Takeda et al., 2022). Decrease in lactate production and accumulation in the skeletal muscles during exercise were reported in several studies (Bonen et al., 1979; Favier et al., 1986; Crowley et al., 1996) and can be a result of the increase in lactate clearance (Donovan & Brooks, 1983). The mechanism of LPS toxicity is very complex involving changes in lipid metabolism, sulfur metabolism and TCA cycle (Dai et al., 2016). Alanine was significantly reduced in plasma and BALF after LPS. The enhancement of hepatic alanine uptake following LPS exposure indicates a metabolic adaptation where the liver increases its utilization of amino acids to support energy production and immune responses during inflammation (Lund et al., 1999). Histidine was significantly reduced in plasma after LPS. A study found that serum histidine levels were significantly decreased in mice with chronic obstructive pulmonary disease (COPD) induced by elastase and LPS. Histidine supplementation improved lung function and reduced inflammation in these models, indicating a protective role against LPS-induced damage (Tian et al., 2021). Animal studies are useful tool to investigate respiratory inflammatory responses, and in particular, the OVA-sensitization and challenge protocol is utilized routinely to study experimental allergic asthma (Warren et al., 2019). Metabolomic study of asthmatic children revealed that histidine, glutamine, methionine, valine, lysine, and proline are associated with allergic sensitization (Chiu et al., 2020). In our study, plasmatic valine has been elevated after OVA sensitization. The same was true for plasma leucine, isoleucine, and 3-hydroxy-butyrate in OVA sensitized animals. 3-hydroxy-butyric acid is synthesized in the liver from acetyl-CoA and is a metabolite of fatty acids and ketogenic amino acids, such as leucine and isoleucine (Manoli & Venditti, 2016). Hydroxy acids were strongly correlated with plasma leucine and valine levels. The accumulation of the hydroxy acids during ketoacidosis may be caused by the derangement of the metabolism of leucine, isoleucine, and valine (Chiu et al., 2020). BCAAs: leucine, isoleucine, and valine, are three of the nine essential amino acids and account for 14% of the total amino acids in skeletal muscle. They share common membrane transport systems and enzymes for their transamination and irreversible oxidation. They can be glucogenic (valine), ketogenic (leucine and isoleucine) or both (isoleucine), since their end products, succinyl-CoA and/or acetyl-CoA can enter the Krebs cycle for energy generation and gluconeogenesis or act as precursors for lipogenesis and ketone body production through acetyl-CoA and acetoacetate (Harper et al., 1984). BCAAs metabolism may impact allergic responses to food and allergy-related outcomes (Chiu et al., 2020). Gluconeogenesis increases during inflammation to meet the heightened demand for glucose needed for immune responses and tissue repair (Shah & Wondisford, 2023). In our study, ovalbumin-sensitized animals showed elevated glucose levels in plasma, indicating an increased energy requirement due to the inflammatory response. Phenylalanine also increased after OVA sensitization. Phenylalanine is potentially important since uncontrolled phenylketonuria is associated with increased plasma immunoglobulin E and atopic dermatitis (Riva et al., 1994). Citrate, elevated in BALF after OVA exposure, plays a dual role in inflammation. It serves as a substrate for the production of pro-inflammatory molecules such as prostaglandin E_2_ (PGE_2_), nitric oxide (NO), and reactive oxygen species (ROS). Additionally, citrate provides precursors for itaconate, which acts as a negative regulator of inflammation by activating anti-inflammatory pathways (Infantino et al., 2019). In our study, we observed that different metabolites were significantly altered following ovalbumin and LPS sensitization. LPS resulted in a substantial reduction in metabolite levels, suggesting that LPS may lead to metabolite depletion, a finding supported by other studies (Corrigan et al., 2014; Dai et al., 2016; Piirsalu et al., 2020). In contrast, ovalbumin sensitization resulted in a significant increase in specific metabolites, indicating that it activates distinct metabolic pathways compared to LPS. This finding was further supported by the comparison of metabolite levels measured in LPS and ovalbumin samples, which showed that metabolite levels were significantly lower in the LPS samples compared to the OVA samples. Conversely, creatine levels were higher in the LPS samples of plasma than in ovalbumin samples. The more pronounced increase in plasma creatine levels in LPS samples compared to OVA samples is likely due to the stronger systemic inflammatory response accompanied by LPS-induced liver or renal damage (Chan et al., 2020; Gao et al., 2021). The fact that lung inflammation can arise from various etiologies makes distinguishing between the allergic and bacterial subtypes highly significant. We evaluated the ability of circulating and BALF metabolites to differentiate between these two subtypes. Using blood plasma metabolite levels as input variables, the random forest algorithm achieved near-perfect discrimination (AUC = 0.978, oob = 0). A slightly lower performance was observed with BALF metabolite levels, though the results were still promising (AUC = 0.945, oob = 2/13). The discriminatory power of the system could be amplified by including both, BALF as well blood plasma metabolites. As expected, the system performed also almost ideally with AUC = 0.998 and oob = 0 (for more details see Suppl. Tab. S4). As metabolomics covers the relatively small size of the number of endogenous molecules related to the number of genes, RNA species or proteins, it is obvious that in the identification of potential low molecular biomarkers some overlap between different pathologies can occur. As reported (López-López et al., 2018), we cannot define the metabolites as biomarkers only based on a ROC, without a validation. To create ROC curve, we used RF algorithm that is not known to overfit the data. On the contrary, RF algorithm picks up two-third data for training and rest for testing for regression and almost 70% data for training and rest for testing during classification to overcome the training and testing on the same data. Although this approach does not replace the need for robust validation with larger sample sets, it can yield encouraging results in exploratory studies. In this study, control animals were not administered sterile saline during sensitization periods, which may have introduced variability between control and experimental groups. As a result, there were minor age differences between the groups (4 days for LPS vs. controls and 14 days for ovalbumin vs. controls), which reduced their comparability. While these differences were assumed to be minimal, they represent limitations of our study design. Future studies should ensure consistent experimental conditions across all groups, including vehicle administration and age matching, to enhance reliability.

## Conclusion

Metabolomics revealed distinct metabolic profiles in plasma and bronchoalveolar lavage fluid of LPS- and OVA-sensitized guinea pigs, identifying potential biomarkers to differentiate between bacterial and allergic inflammation. OVA sensitization was manifested by elevated ketone bodies accompanied by mild hyperglycaemia linked with an increase in blood plasma levels of BCAAs against those found in control animals. LPS-sensitization, when compared with controls, led to the depletion of circulating BCKAs, alanine and glutamine interconnected in the body’s metabolism, especially in amino acid catabolism and nitrogen balance, and together with alterations in lactate and pyruvate level affecting glycolysis, gluconeogenesis and energy gaining processes. The comparison of LPS and OVA sensitizations largely reflected characteristic metabolic features of both individual models. Based on metabolic data from blood plasma as well as from BALF, the successful discrimination between both models, OVA and LPS, which are inducing acute systemic and lung inflammation is attainable.

## Supplementary Information

Below is the link to the electronic supplementary material.

Supplementary file1 (DOCX 386 KB)

## Data Availability

NMR spectra, as well as evaluated levels of metabolites are available on request: eva.baranovicova@uniba.sk.
