# Supplementary material for: Metabolic profiling in experimental guinea pig models of bacterial and allergic inflammation
Source: Metabolomics. 2025 Mar 23;21(2):43. doi: 10.1007/s11306-025-02239-x (PMC11930882; doi:10.1007/s11306-025-02239-x)

***Metabolic profiling in experimental guinea pig models of bacterial and allergic inflammation***

*Hanusrichterova J^1^, Baranovicova E^1^, Barosova R^2^, Kolomaznik M^1^, Mikolka P^2^, Kosutova P^1^, Mokra D^2^, Mokry J^3^, Calkovska A^2^

***Online supplement***

***Metabolomics***

^1^Biomedical Centre Martin, Jessenius Faculty of Medicine in Martin, Comenius University in Bratislava, Martin, Slovakia

^2^Department of Physiology, Jessenius Faculty of Medicine in Martin, Comenius University in Bratislava, Martin, Slovakia

^3^Department of Pharmacology, Jessenius Faculty of Medicine in Martin, Comenius University in Bratislava, Martin, Slovakia

***Corresponding author:*** Juliana Hanusrichterova, Biomedical Centre Martin, Jessenius Faculty of Medicine in Martin, Comenius University in Bratislava, Mala Hora 11161/4D, 036 01 Martin, Slovakia. E-mail: topercerova4@uniba.sk; ORCID number: 0009-0009-2781-2264.

**Suppl. Tab. S1** Chemical shifts (in ppm), J couplings (in Hz) and multiplicities (s - singlet, d - doublet, t - triplet, q - quartet, m - multiplet, dd - doublet of doublets, dq - doublet of quartets) for the pool of metabolites identified in blood plasma and BALF, D_2_O pH 7.4, 600 MHz Bruker Avance with cryoprobe, signals marked with # were not suitable for quantitative evaluation

|  |  |
| --- | --- |
| **metabolite** | **NMR peak assignment** |
| #threonine | 1.34 (d), 3.56 (d; J= 4.9), 4.26 (m) |
| 2-ketoisovalerate (2-ketovaline) | 1.11 (d; J=7.1), 3.01(dq) |
| 2-oxoisocapronate (2-ketoleucine) | 0.94 (d; J=6.6), 2.11 (m), 2.61 (d; J=7.0) |
| 3-hydroxybutyrate | 1.20 (d; J= 6.23 Hz), 2.31 (m, J= 14.4, 6.2), 2.41 (m, J= 14.4, 6.2), 4.16 (m) |
| 3-methyl-2-oxo-valerate (2-ketoisoleucine) | 0.90 (t; J=7.5), 1.10 (d; J=6.7) |
| acetate | 1.92 (s) |
| alanine | 1.48 (d; J=7.30), 3.78 (q, J = 7.23) |
| citrate | 2.54 (d; J = 15.1), 2.67 (d; J = 15.1) |
| creatine | 3.04 (s), 3.94 (s) |
| creatine | 3.03(s), 3.92(s) |
| creatinine | 3.05 (s), 4.07 (s) |
| creatinine | 3.06 (s), 4.07(s) |
| glucose | 3.23 (m), 3.40 (m), 3.46 (m), 3.52 (dd, J= 3.8, 9.8), 3.78 (m, J= 5.4), 3.82 (m, 10.8, 5.4), 3.89 (dd, J = 10.8, 9.8), 4.64 (d, J= 7.6, 5.23 (d, J= 1.6) |
| glutamine | 2.12 (m; J=6.8, 6.2), 2.15 (m, J=6.8, 6.2), 2.44 (m, J= 14.6, 6.8), 2.48 (m, J= 14.6, 6.8), 3.77 (dd, J=6.2) |
| glycine | 3.561 (s) |
| histidine | 7.07 (s), 7.80 (s) |
| isoleucine | 0.94 (t; J = 7.5), 1.01 (d; J = 7.0), 3.68 (d; J = 4.2) |
| lactate | 1.33 (d; J = 7.0), 4.12 (q; J = 7.0) |
| leucine | 0.96 (d; J = 6.2), 0.97 (d; J = 6.1), 1.68 (m), 1.72 (m), 1.75(m) |
| lipoprotein fraction | 0.82-0.93 (m), 1.20-1.37 (m) |
| lysine | 1.33 (d), 3.58 (d; J=4.9), 4.25 (m) |
| myo-Inositol | 3.28 (t; J= 9.42), 3.54 (dd; 10.03, 3.0), 3.64 (dd; J=9.75), 4.07 (dd; J= 2.89) |
| phenylalanine | 3.13 (m), 3.28 (m, J= 15.4, 6.3), 7.34 (d; J=7.5), 7.38 (t; J=7.4), 7.44 (t, J=7.1, 3.5) |
| proline | 1.46 (m), 1.50 (m), 1.73 (m), 1.89 (m), 1.93 (m), 3.03 (t; J=7.6) |
| pyruvate | 2.38 (s) |
| pyruvate | 2.367 (s) |
| succinate | 2.404 (s) |
| tryptophan | 7.21 (t), 7.30 (td, J=7.3, 1.1), 7.33 (s), 7.56 (d; J = 8.1), 7.74 (d; J = 8.0) |
| tyrosine | 3.05 (dd, J = 14.7, 6.8), 3.20 (dd, J= 14.7, 5.1), 3.93 (dd, J= 5.1, 7.7), 6.91 (d; J=8.5), 7.20 (d; J=8.5) |
| valine | 0.99 (d; J=7.1), 1.04 (d; J=7.1), 2.27 (m), 3.61 (d; J=4.4) |

**Suppl. Tab. S2** Statistical evaluation of relative plasma metabolite concentrations between the control and OVA or LPS-sensitized animals and LPS and OVA. In OVA/Control and LPS/Control, Change % = (post-treatment median – control median / control median) x 100. In LPS/OVA, Change % = (LPS median – OVA median / OVA median) x 100. Data compared with non-parametric Mann-Whitney U test. Significant changes with a p<0.05 are listed along with percentage change derived from medians

| ***Blood plasma*** |  |  |  |
| --- | --- | --- | --- |
|  |  |  |  |
|  |  |  |  |
| ***Compared groups*** | ***Metabolites*** | ***p value*** | ***Change (%)*** |
|  |  |  |  |
|  |  |  |  |
| ***OVA/Control*** | *Valine* | *0.045* | *21.427* |
|  | *Leucine* | *0.045* | *30.644* |
|  | *Isoleucine* | *0.045* | *22.785* |
|  | *Phenylalanine* | *0.045* | *23.568* |
|  | *Glucose* | *0.019* | *29.890* |
|  | *3-hydroxy-butyrate* | *0.019* | *44.622* |
|  |  |  |  |
|  |  |  |  |
| ***LPS/Control*** | *Lactate* | *0.021* | *-33.732* |
|  | *Alanine* | *0.002* | *-35.439* |
|  | *Pyruvate* | *0.0003* | *-74.730* |
|  | *Glutamine* | *0.0006* | *-40.749* |
|  | *Ketoleucine* | *0.004* | *-26.340* |
|  | *Ketoisoleucine* | *0.021* | *-31.338* |
|  | *Ketovaline* | *0.040* | *-29.178* |
|  | *Histidine* | *0.0003* | *-57.964* |
|  |  |  |  |
|  |  |  |  |
| ***LPS/OVA*** | *Lactate* | *0.030* | *-44.327* |
|  | *Alanine* | *0.003* | *-38.153* |
|  | *Glucose* | *0.003* | *-19.716* |
|  | *Leucine* | *0.048* | *-28.252* |
|  | *Pyruvate* | *0.003* | *-43.069* |
|  | *3-hydroxy-butyrate* | *0.048* | *-29.981* |
|  | *Ketoleucine* | *0.003* | *-30.319* |
|  | *Ketoisoleucine* | *0.010* | *-40.274* |
|  | *Ketovaline* | *0.048* | *-35.733* |
|  | *Creatine* | *0.003* | *38.630* |
|  | *Creatinine* | *0.005* | *-49.850* |
|  | *Proline* | *0.030* | *-25.726* |
|  | *Histidine* | *0.003* | *-56.892* |
|  | *Tryptophan* | *0.010* | *-45.382* |

**Suppl. Tab. S3** Statistical evaluation of relative BALF metabolite concentrations between the control and OVA or LPS-sensitized animals and LPS and OVA. In OVA/Control and LPS/Control, Change % = (post-treatment median – control median / control median) x 100. In LPS/OVA, Change % = (LPS median – OVA median / OVA median) x 100. Data compared with non-parametric Mann-Whitney U test. Significant changes with a p<0.05 are listed along with percentage change derived from medians

| ***Bronchoalveolar lavage fluid*** |  |  |  |
| --- | --- | --- | --- |
|  |  |  |  |
|  |  |  |  |
| ***Compared groups*** | ***Metabolites*** | ***p value*** | ***Change (%)*** |
|  |  |  |  |
|  |  |  |  |
| ***OVA/Control*** | *Citrate* | *0.048* | *19.457* |
|  |  |  |  |
|  |  |  |  |
| ***LPS/Control*** | *Alanine* | *0.006* | *-16.839* |
|  | *Valine* | *0.040* | *-11.076* |
|  | *Pyruvate* | *0.0003* | *-56.490* |
|  | *Succinate* | *0.004* | *-30.016* |
|  |  |  |  |
|  |  |  |  |
| ***LPS/OVA*** | *Alanine* | *0.018* | *-30.350* |
|  | *Glutamate* | *0.030* | *-60.542* |
|  | *Tyrosine* | *0.005* | *-25.832* |
|  | *Succinate* | *0.018* | *-28.544* |
|  | *Glycine* | *0.018* | *-27.192* |
|  | *Proline* | *0.030* | *-34.572* |

**Suppl. Tab. S4** Statistical parameters for multivariate supervised PCA and PLS-DA method, and discriminatory performance parameters from cross-validated random forest algorithm

**Suppl. Fig.** **S1** Total white blood cell (WBC) count and differential WBC count in BALF of LPS (Control LPS n=8; LPS n=7) and OVA-sensitized guinea pigs (Control OVA n=8; OVA n=5). Counts of WBC, neutrophils (NEUT), lymphocytes (LYMPH), monocytes (MONO), eosinophils (EO), and basophils (BASO) were expressed in number of cells × 10^9^/L (evaluated by an automatic hematology analyzer Sysmex XT-2000i, Landskrona, Sweden). Data are expressed as mean ± SEM. Control and sensitized groups were compared with unpaired Student t-test if data were normally distributed or Mann-Whitney U test if data failed Shapiro-Wilk normality test. The white blood cell count data were processed and statistically evaluated in the software GraphPad Prism version 8.0.1 (GraphPad Software, USA). Statistical significance *p<0.05; **p<0.01


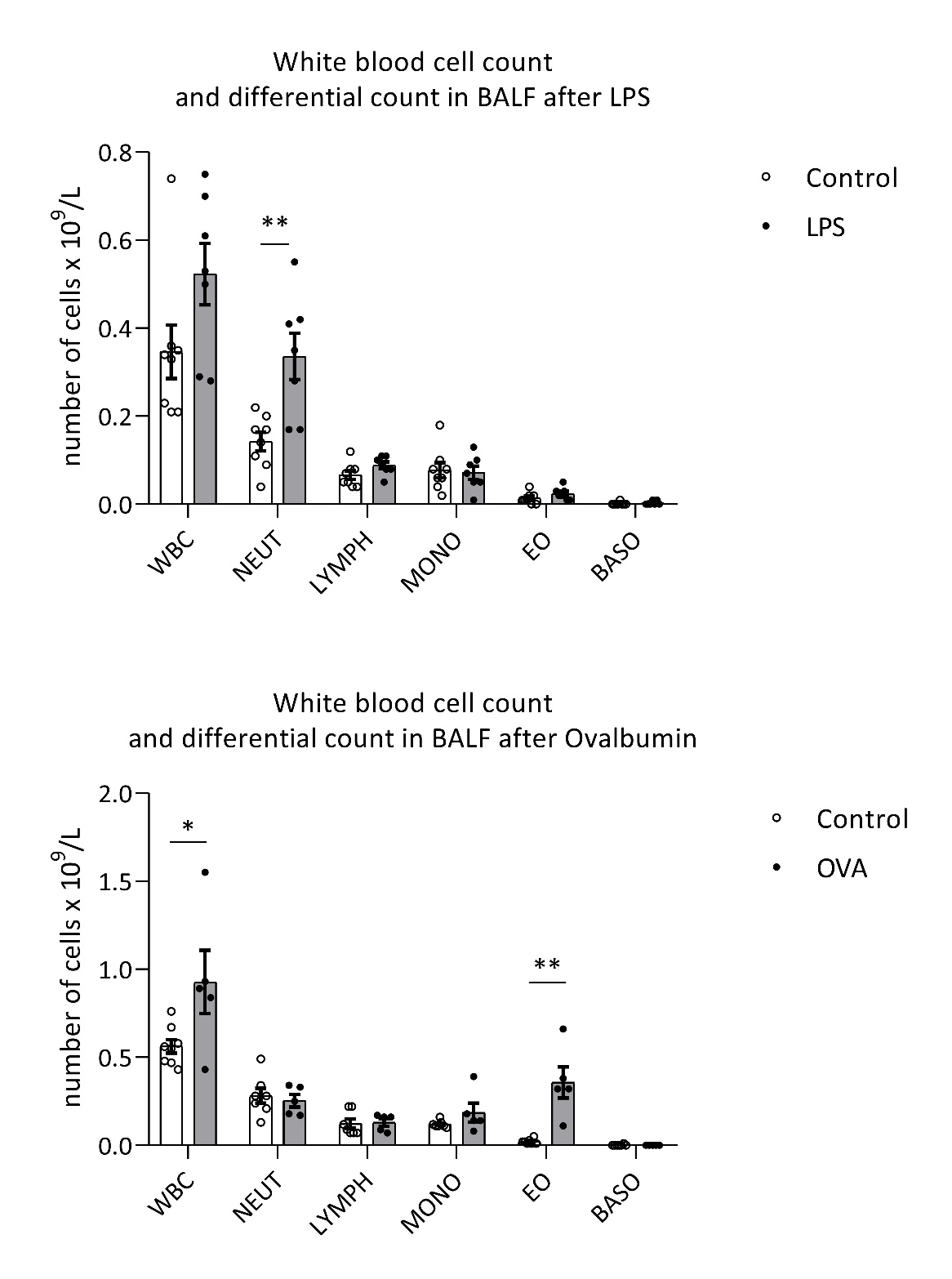


**Suppl. Fig. S2** PLS-DA analysis of plasmatic metabolome (A) and BALF metabolome (B) for groups LPS control and LPS, OVA control and OVA and LPS and OVA with 95 % confidence ellipse (ellipse that defines the region that contains 95 % of all samples). As input variables, relative levels of blood plasma and BALF metabolites were used


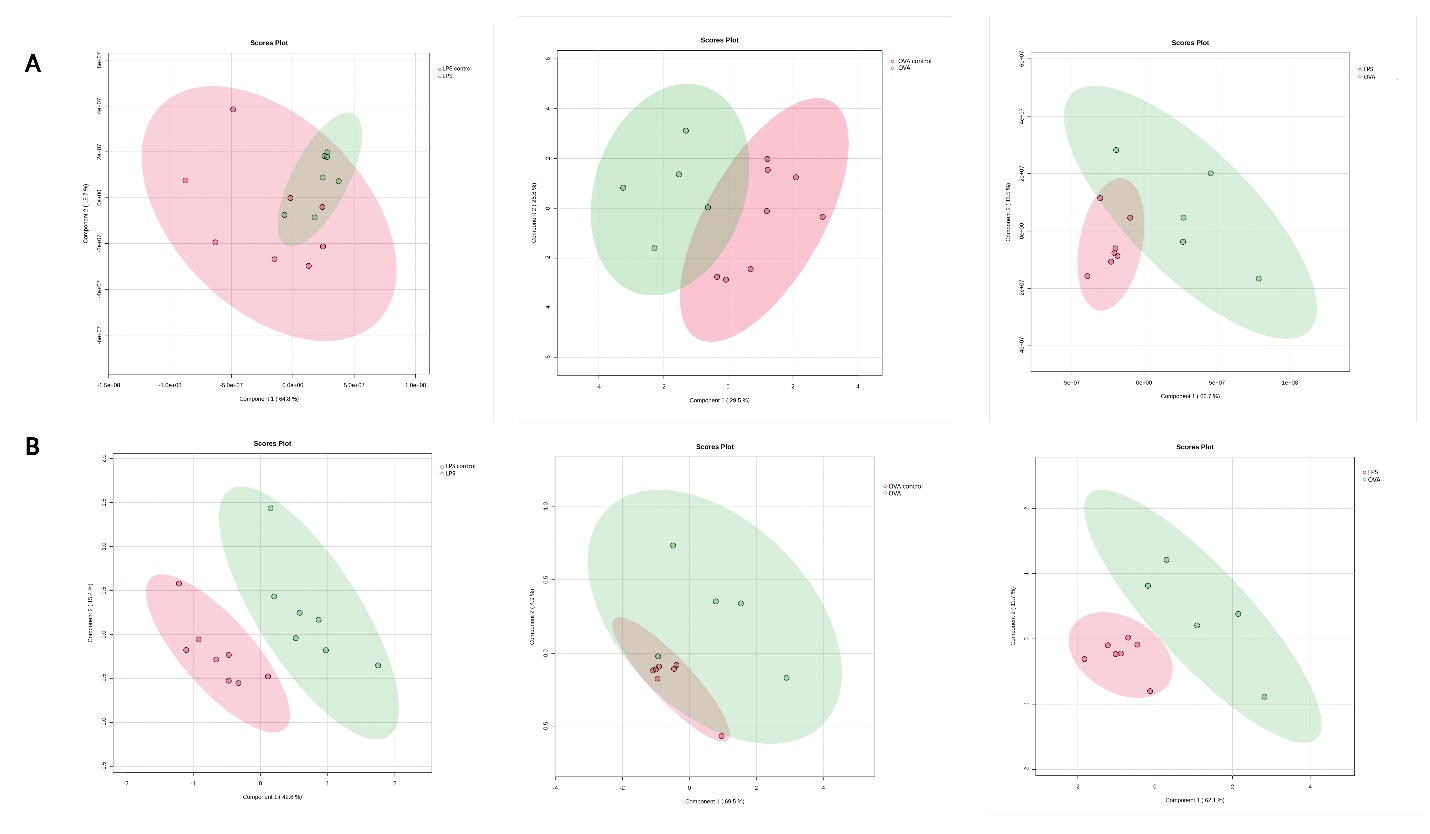


**Suppl. Fig. S3** PLS-DA analysis of LPS (red) vs OVA (green) comparison when all metabolic data (Plasma and BALF) were used with 95 % confidence ellipse (ellipse that defines the region that contains 95 % of all samples). As input variables, relative levels of blood plasma and BALF metabolites were used


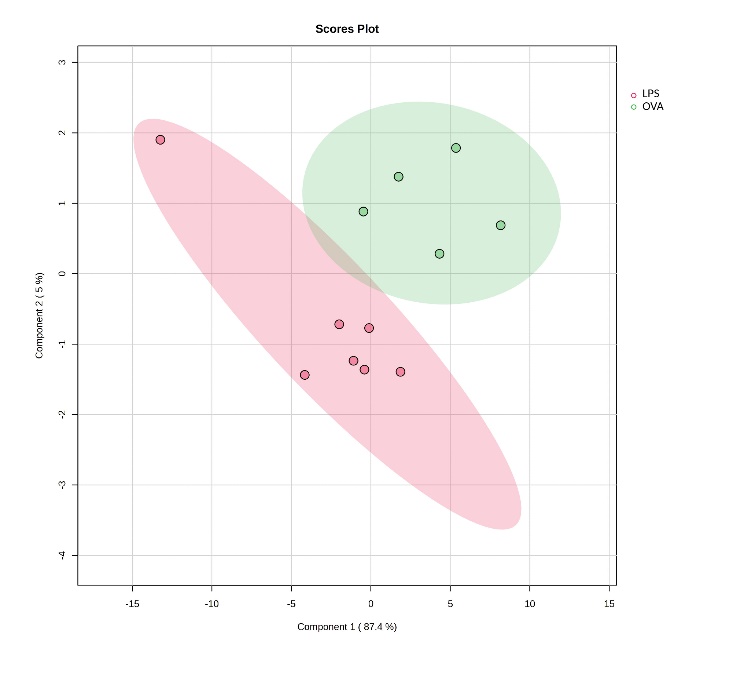

Supplement: Supplementary file 1 — Supplementary file1 (DOCX 386 KB) [file 11306_2025_2239_MOESM1_ESM.docx]
